# Bioinformatic analysis of smoothelin family members supports tissue‐specific functions of unique C‐terminal calponin homology domains

**DOI:** 10.14814/phy2.15844

**Published:** 2023-11-13

**Authors:** Dhruv Garg, Steven A. Fisher

**Affiliations:** ^1^ Marriotts Ridge High School Baltimore Maryland USA; ^2^ Departments of Medicine (Cardiology) and Physiology University of Maryland School of Medicine Baltimore Maryland USA; ^3^ Baltimore Veterans Affairs Medical Center Baltimore Maryland USA

## Abstract

Smoothelins are cytoskeletal proteins with a single C‐terminal calponin homology domain type 2 (CHD2). Little is known about the significance of variation in SMTN CHD2 domains, addressed here through analysis of public databases. A conserved 152 nt penultimate constitutive exon present in all SMTNs encodes helices II‐IV of CHD2 with high identity (nt/aa 63/65%). Variable CHD2s of SMTN (helices IV‐VI) are generated by alternative splicing of 165 nt exon E20. E20 and the CHD2 it encodes have high homology with the terminal constitutive exon of SMTNL1 (E8; nt/aa 72/75% identity). Unique to these CHD2 variants are a conserved extended nine amino acid C‐terminal tail containing KTKK ubiquitination motifs. When E20 of SMTN is skipped (SMTN E20−), constitutive terminal E21 codes for helices IV‐VI of CHD2. SMTN E21 has high identity with the terminal exon of SMTNL2 (E8; nt/aa 75/81% identity of aligned sequences) except for coding for a unique extended C‐terminus (24 nt; 8aa) conserved only in mammals. SMTN isoform expression is tissue‐specific: SMTNE20− and SMTNE20+ are highly expressed in SMC and non‐muscle cells, respectively, while SMTNL1 + 2 are highly expressed in skeletal muscle cells. Tissue‐specific expression of SMTN CHD2s with unique helices IV‐VI suggest tissue‐specific functions that require further study.

## 1 INTRODUCTION

Alternative exon usage (AEU) is common to most genes (Merkin et al., 2012; Wright et al., 2022) and a major driver of smooth muscle phenotypic diversity (Fisher, 2010, 2017). Yet for only a few of the many protein isoforms generated by AEU is the functional significance known or even hypothesized. Smoothelin (SMTN) is a cytoskeletal protein highly enriched in differentiated smooth muscle cells (SMCs) (Rensen et al., 2002; van der Loop et al., 1996) (reviewed in Murali & MacDonald, 2018). The C‐terminus of SMTN contains a single calponin homology domain type 2 (CHD2) with two isoforms generated by alternative splicing of a 165 nt penultimate exon (E20) (Kramer et al., 2001; Rensen et al., 2002) (reviewed in van Eys et al., 2007). The CHD was originally described in analysis of the Vav guanine nucleotide exchange factor (Castresana & Saraste, 1995) and subsequently found in more than 100 mostly cytoskeletal proteins usually in the context of an actin‐binding domain (Yin et al., 2020). The CHD is ~100 residues forming 6 α‐helices with connecting linker sequences. However, the function of the CHD within cytoskeletal proteins is more diverse than actin‐binding. In fact, it is thought that a single CHD, as in SMTN, may be insufficient to mediate actin binding (Gimona et al., 2002), though this remains unsettled and other binding partners and functions in cell signaling have been proposed (Yin et al., 2020). Ironically the specific function of calponin and its namesake single CH3 domain in smooth muscle has yet to be conclusively defined (Liu & Jin, 2016; Yin et al., 2020).

SMTN E20 is one of the most highly regulated AEs in SMCs (Llorian et al., 2016). Splicing of E20 undergoes a switch from 70% to 15% E20 inclusion during postnatal maturation of mouse mesenteric arterial smooth muscle, with a similar though less well‐defined switch during prenatal maturation of mouse bladder smooth muscle (Zheng et al., 2015). When mature mouse aorta and bladder SMCs de‐differentiate and proliferate in vitro, SMTN mRNA shifts back to the embryonic/ non‐muscle cell pattern, from 16% to 18% E20 inclusion to 98% and 68% inclusion, respectively (Llorian et al., 2016). This is similar to another AE we have studied for many years, E24 of Mypt1, except that the pattern is reversed, with inclusion in differentiated SMCs (Payne et al., 2006) and a switch to skipping in proliferative SMCs (Llorian et al., 2016) and in disease models (Payne et al., 2004; Zhang & Fisher, 2007). In the case of Mypt1 E24 splice variants, we have proposed that alternative splicing of E24, by toggling expression of a C‐terminal LZ motif, sets the sensitivity for activation of the enzyme by cGMPkinase and thus NO/cGMP vasodilator signaling (reviewed in Brozovich et al., 2016; Fisher, 2017). In contrast, the functional significance of alternative CHDs generated by alternative splicing of SMTN and multiple gene family members (SMTN, SMTNL1 + 2) are unknown. Here we use public databases and computational approaches to analyze sequences, predict structures, and analyze patterns of SMTN isoforms and family members, to learn as much as we can about SMTN family isoforms with existing datasets.

## 2 METHODS

Ensembl and UniProt websites were used to download protein and nucleotide sequence data. Sequences evaluated in this study include ENSMUST00000170588.8 (Smtn‐210; E20+), ENSMUST00000075118.10 (Smtn‐203; E20−), ENSMUST00000028471.6 (Smtnl1‐201), ENSMUST00000050226.7 (Smtnl2‐201). The UCSC Genome Browser was used to determine phylogenetic conservation. Clustal Omega's Multiple Sequence Alignment Tool was used to align SMTN protein sequences. AlphaFold and AlphaFold2 were used to determine the secondary structures of the SMTN family proteins. AlphaFold2 is a protein structure predictor tool, currently in beta testing, and was primarily used when the structure of a protein had not been determined by alphafold. NCBI's Conserved Domain Search was used to identify the precise location and presence of the CHD. AlphaFold's sequence data were then used to map the exact amino acid sequence of every alpha‐helix in the SMTN family's CHD. Human expression data for SMTN family mRNAs was downloaded from GTEx Portal. Expression data for SMTN family mRNAs in single cells from mouse brain and lung vasculature were downloaded from the Betsholtz lab website (Vanlandewijck et al., 2018).

## 3 RESULTS

SMTN splice variant isoforms code for alternative CHD sequences with high phylogenetic conservation.

**FIGURE 1:**
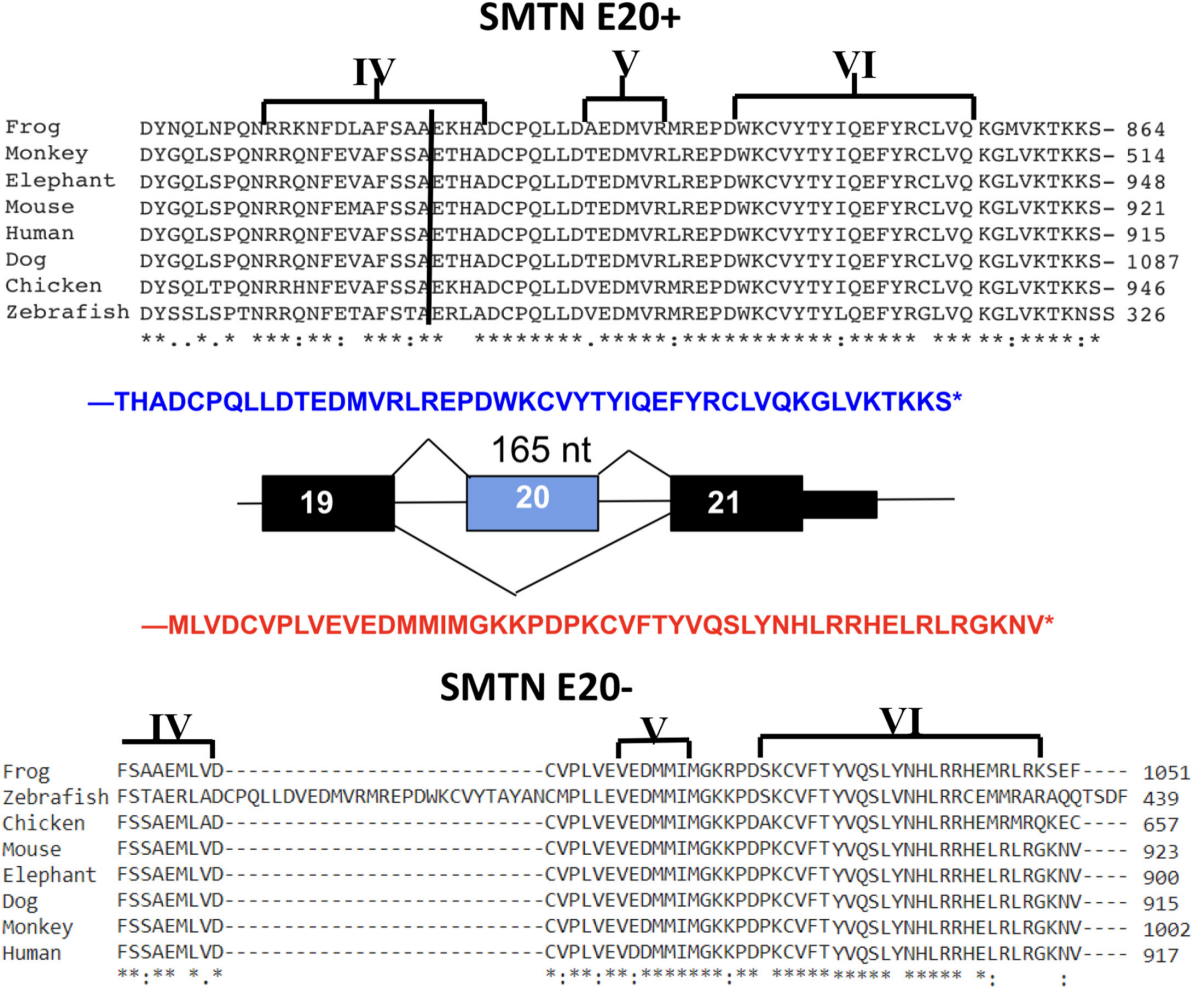
Alternative splicing of SMTN E20 and phylogenetic conservation of SMTN isoforms. Alternative exon 20 (E20) of mouse SMTN undergoes cassette‐type alternative splicing to generate isoforms with unique C‐terminal CHDs sequences as shown. E20 contains an internal stop codon resulting in a shorter protein. SMTN C‐terminal CHD2 domains helices IV‐VI encoded by SMTNE20+ and E20− across vertebrates were aligned using CLUSTAL. These are highly conserved with the exception of the terminal eight amino acids encoded by SMTNE20‐, conserved in mammals but not non‐mammals, and a 28 amino acid insert in the linker between loops IV and V of zebrafish. E, exon; SMTN, smoothelin. * identical; strongly similar; weakly similar.

SMTN E20+ and SMTN E20− splice variants each code for unique C‐terminal CHDs that are highly conserved phylogenetically in mammals, birds, amphibians, and fish (Figure 1). The unique amino acid sequences encoded by the E20+ variant (non‐muscle) begin toward the end of helix IV of the CHD (Figure 1) and continues to the termination codon encoded within E20 48 amino acids later. The few amino acids that are not conserved in this stretch are due to variation in zebrafish and to a lesser extent the frog. Skipping of E20 (SMTN E20−; SMC‐specific) results in E21 encoding the terminal CHD that extends to the termination codon 50 amino acids later. This also is highly phylogenetically conserved except for (1) in zebrafish a 27 amino acid insert in the linker domain between helices IV and V (2) the C‐terminal eight amino acids, which are well conserved in mammals only.

### 3.1 Calponin homology domains in SMTN family members

**FIGURE 2:**
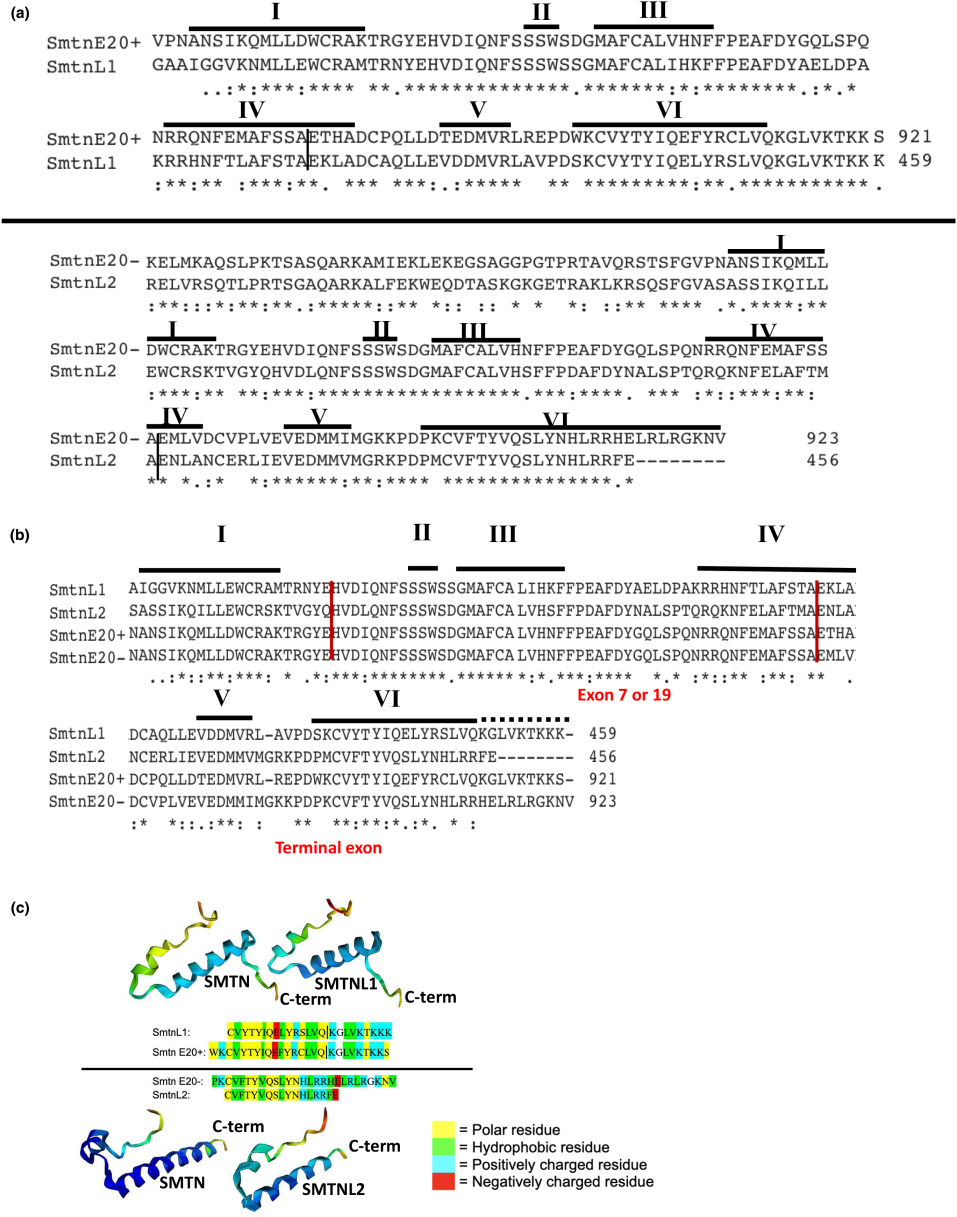
Alignments of SMTN, SMTNL1, and SMTNL2 CHDs and predicted secondary structures (a) Amino acid alignments of mouse CHDs encoded by SMTNE20+ and SMTNL1 and SMTNE20− and SMTNL2 using CLUSTAL. The vertical line indicates exon–exon boundary. Horizontal lines indicate the CHD helix with the number indicated above the line. CHD2s of SMTNE20+ and SMTNL1 are nearly identical, including in variable helices V‐VI and C‐terminal tail. Similarly, CHD2s of SMTNE20− and SMTNL2 are nearly identical, including in variable helices V‐VI with the exception of an 8 amino acid extension at the C‐terminus of SMTNE20−. (b) Alignment of mouse SMTNL1/2 and SMTNE20± CHD2s. Of note is the high conservation of helices II‐III and adjacent linker sequence. (c) CHDs' variable helices V‐VI predicted structures. Amino acid sequences as shown were input into alphafold and alphafold2 and predicted secondary structures were obtained. Amino acid sequences are color‐shaded according to properties as polar, hydrophobic, or positively or negatively charged. Vertical line indicates end of helix VI. Sequences downloaded for evaluation include ENSMUST00000170588.8 (Smtn‐210; E20+), ENSMUST00000075118.10 (Smtn‐203; E20−), ENSMUST00000028471.6 (Smtnl1‐201), ENSMUST00000050226.7 (Smtnl2‐201). Coloring of ribbon structures is according to confidence of prediction: red = <50%, yellow = 60%, green = 70%, light blue = 80%, dark blue = >90%.

We next aligned the unique CHDs encoded by SMTN E20+/− with SMTN family members SMTNL1 and SMTNL2 (Figure 2). Interestingly the mouse SMTN E20+ encoded CHD has high identity with the CHD of mouse SMTNL1. The mouse SMTN E20− encoded CHD has high identity with the CHD of mouse SMTNL2 (Figure 2a). An exception was the C‐terminal 8 amino acids, absent from SMTNL2 and also not conserved in non‐mammalian SMTN (Figure 1) An alignment of the four mouse SMTN isoforms encoded by SMTNE20+; SMTNE20−; SMTNL1 and SMTNL2 shows the similarities and differences in the C‐terminal CHD amino acid sequences (Figure 2b). The highly conserved sequence in and around Loops II‐III of SMTNx CHD and a subset of other CHD2‐containing cytoskeletal proteins was previously noted (Figure 1 in Ishida et al., 2008). There is little homology between SMTN, SMTNL1, and SMTNL2 N‐terminus of the CHDs, not surprising given the highly unstructured nature of this region (reviewed in Murali & MacDonald, 2018).

### 3.2 Analysis of SMTN family members’ CHD sequence and predicted structures

We used alphafold programs to predict the secondary structures of the unique CHD sequences (Figure 2c). The CHDs of SMTNL1 and SMTN E20+ each have 5 turns in helix VI followed by an unstructured nine amino acid tail. In contrast, SMTN E20− has 7 turns in helix VI without an extended C‐terminus tail. SMTNL2 C‐terminus has the strongest sequence homology to SMTN E20−, but because of a truncated C‐terminus only has 5 turns in loop 6. The amino acid compositions of the C‐termini of the four isoforms are broadly similar with approximately similar proportions of polar, hydrophobic, positively and negatively charged residues. For each isoform, the proximal portion of helix VI is mostly polar and hydrophobic residues, while the very C‐terminus is predominately positively charged residues. Interestingly a (KTKK [S/K]) motif is present only in the extended cytoplasmic tails of SMTN and SMTNL1 and may function as a ubiquitination motif. The extended α‐helix of SMTN E20− is unique to this isoform and could indicate a unique function in SMCs where it is uniquely expressed.

### 3.3 Expression of SMTN family members

**FIGURE 3:**
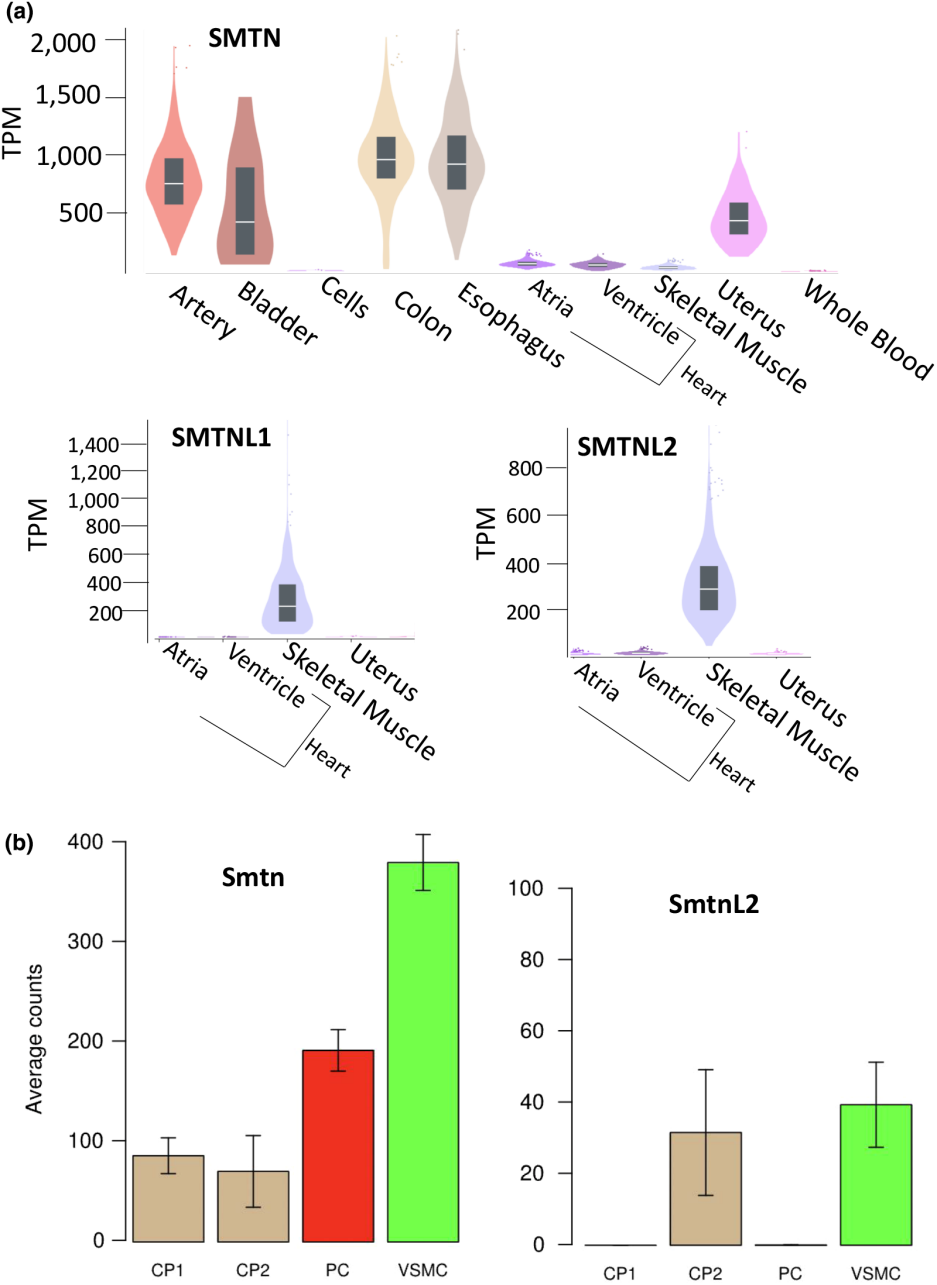
Expression of SMTNx mRNAs in human and mouse tissues and cells. (a) Median read counts (transcripts per million) from indicated human tissues from the Gtexportal (www.GtexPortal.com) shown on the y‐axis starting at zero. SMTN is highly expressed in smooth muscle‐containing vascular and visceral tissues and at low levels in striated muscle and non‐muscle tissues. SMTNL1 + 2 are highly expressed in skeletal muscle and at low levels in other tissues. Data shown are violin plots with average values from tissues obtained from *n* = 20–584 individuals. These are unpurified samples. Samples shown include tibial artery (artery) and Epstein‐Barr virus‐transformed lymphocytes (cells). (b) The Betsholtz laboratory database (www.betsholtzlab/vascularsinglecells/database.html) was interrogated for expression of SMTN family members. SMTN is most highly expressed in mouse lung vascular smooth muscle cells, at intermediate levels in lung pericytes and low levels in non‐vascular cells. SMTNL2 was detected at low levels in a few vascular cell types (note differing y‐axis) and at intermediate levels in vascular fibroblastic cells (not shown). Data are from single cells expressing different cell type‐specific fluorescent reporters purified from mouse lung vessels (Vanlandewijck et al., 2018). Data are shown here as average counts on the y‐axis and cell type on the x‐axis (*n* = 2–3 mice per cell type). Raw data for counts per cell can be found on the Betsholtz laboratory website. CP, cartilage perichondrium; PC, pericytes; VSMC, vascular smooth muscle cells.

As expression is the sine qua non of function, we examined public databases to examine mRNA levels of SMTN family members in human tissues (GtexPortal) and single cells of mouse vessels (Betsholtz lab website). Consistent with the use of SMTN as a marker for SMC differentiation, SMTN is present at high levels in human vascular and visceral smooth muscle‐containing tissues and at low levels in cardiac and skeletal muscle and non‐muscle cells (Figure 3a). In analyses of single cells purified from mouse lung (Figure 3b) vessels, SMTN is highly expressed in vascular SMCs, expressed at intermediate levels in pericytes and at low levels in non‐SMCs. A similar pattern is observed in single cells isolated from brain vasculature (see Betsholtz laboratory website). SMTNL1 + 2 are highly expressed in human skeletal muscle with low‐to‐absent expression in cardiac and uterine tissues (Figure 3a). In the human artery samples—tibial, coronary, and aorta—the median counts of transcripts per million were 0.4–0.6 for SMTNL1 and 0.5–0.8 for SMTNL2, 250–500 fold lower than in the highly expressing skeletal muscle. This pattern of expression in humans is consistent with a prior study showing that SMTNL1 + 2 proteins are undetectable in mouse myoblasts and highly induced during muscle differentiation (Gordon et al., 2013). SMTNL2 mRNA was detected at low levels in mouse lung VSMCs (~1/10 the level of SMTN) and CP2 cells (Figure 3b) and at intermediate levels (~100 counts) in sub‐populations of lung vessel fibroblast‐like cells, while SMTNL1 mRNA was not detected at all.

### 3.4 SMTN family member gene structures and sequence conservation

**FIGURE 4:**
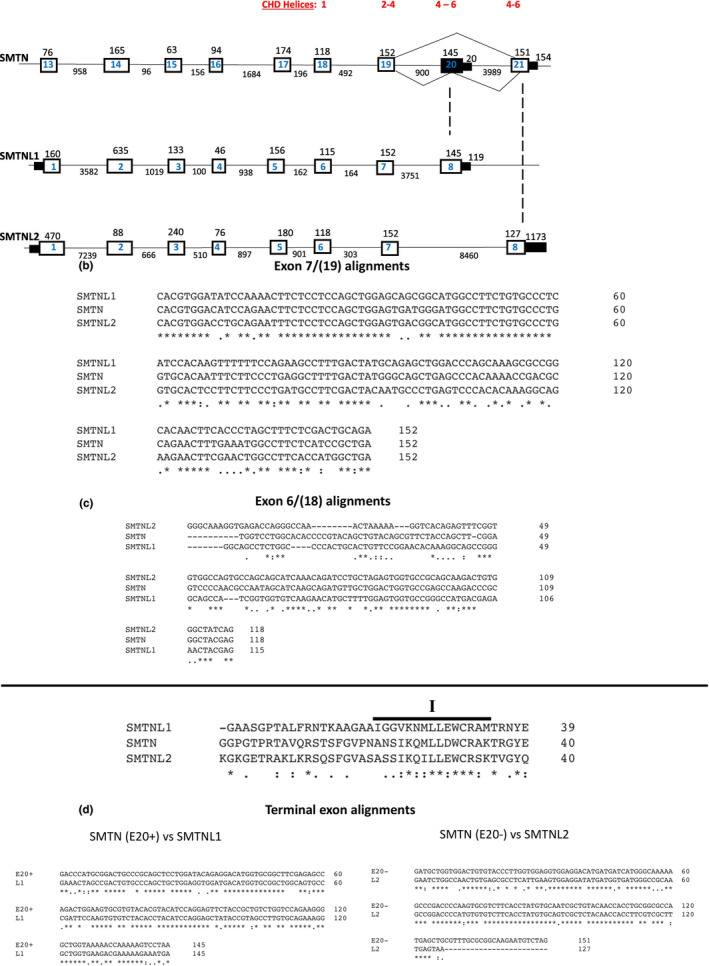
SMTN family member gene exon‐intron structures and alignments. (a) Exon‐intron structures of mouse SMTNx genes were obtained from the UCSC gene and ENSEMBL browsers. They are redrawn here not to scale with exons indicated by boxes and introns by lines. Exon size in nt is indicated above the box and intron size in nt below the line. (b) The 152 nt exon 7 of SMTNL1+ 2 alignment with SMTN E19. (c) Alignment of 118/115 nt E6 of SMTNL2/1, respectively, with 118 nt E18 of SMTN and alignment of amino acid sequences they encode. (d) Alignment of coding sequences of terminal exons of SMTN (E20, E21) with terminal exons (E8) of SMTNL1/2, respectively. The 165 alternative exon 20 of SMTN has 145 nt of coding sequence and a premature stop codon. This is similar to exon 8 of SMTNL1 and encodes highly conserved variable Loops IV‐VI of CHD2. The 151 nt terminal E21 of SMTN encodes the alternative CHD2 domain loops IV‐VI and is most similar to terminal E8 of SMTNL2 excepting a 28 nt extension in the former.

SMTN family member genes are compact and well‐conserved (Figure 4a). Mouse SMTNL1 is the smallest at 10.5 kb. The corresponding portion of the SMTN gene is 11.5 kb. This includes a unique first exon giving rise to the SMTN‐A isoform enriched in visceral SMCs (van Eys et al., 2007). The SMTN gene has a unique 5′ extension of 11 exons over a span of 11.5 kb that generates the SMTN‐B isoform selectively expressed in vascular SMCs. Homologous sequence is not present in SMTNL1/2. Mouse SMTNL2 is the largest at 22.5 kb (excluding non‐homologous SMTN‐A) due to expansion of the first and last introns and an extended 3′ untranslated region (Figure 4a). This extended 3'UTR could be the site for unique regulation by micro‐RNA mediated mRNA de‐stabilization. The invariant penultimate constitutive 152 nt E7 (SMTN E19) (Figure 4a) sequence is highly conserved in SMTNL1/2 (Figure 4b). It codes for highly conserved helices II‐IV of CHD2 present in SMTNx (Figure 2b) and other proteins with highly homologous CHD2s including MICAL, dystrophin/utrophin, plectin, actinin, and others (see Figure 1 of Ishida et al., 2008). The preceding E6 (E18 in SMTN) is nearly identical in size between SMTNx; the 3′ half of the exon coding for helix I of CHD2 is well‐conserved (Figures 2c and 4c). Upstream of E6 the exon‐intron structure is preserved but exon sizes are markedly different (Figure 4a) with poor conservation of sequence (see UCSC genome browser). This is unsurprising given that this codes for the unstructured N‐terminus of SMTNx. Phylogenetic analysis confirms that the four most 3′ exons encoding CHD2 are the essential attributes of SMTNx, as these are invariably present and well‐conserved phylogenetically. In contrast, the first four exons of SMTNL1/2 coding for the N‐terminal unstructured domain are often missing in more evolutionarily distant organisms such as non‐mammalian vertebrates (see UCSC genome browser).

The variable portion of SMTNx CHD2 is in Loops IV‐VI. This is encoded in terminal E8 of SMTNL1 and homologous E20 of SMTN (Figure 4d) with high identity of the nt coding sequence (initial 145 nt; Figure 4b) and the amino acids they encode (Figure 2a). This exon is not present in SMTNL2, perhaps due to its being lost within the expanded terminal intron (~8.4 kb; Figure 4a). Instead, the SMTNL2 terminal E8 is 8.4 kb downstream of E7 and homologous to SMTN E21. There is high conservation of 127 nt coding sequence with the exception of a 24 nt extension of SMTN E21 (Figure 4d) coding for a unique extension of helix VI of CHD2 (Figure 2).

## 4 DISCUSSION

A single C‐terminal CHD2 is the defining feature of SMTN family members. The SMTN CHD2 has two variants composed of fixed Loops I‐III encoded by the highly conserved two most penultimate constitutive exons, and variable loops IV‐VI generated by a variable terminal exon. These two forms of the C‐terminal CHD2 are generated in SMTN via alternative splicing of E20, while each is present in SMTNL (SMTNL1, 2) via homologous constitutively spliced terminal exons. While CHDs present in cytoskeletal proteins are generally involved in actin binding, their function is thought to go well beyond this in serving as a signaling hub, and ironically the specific function of the single CHD3 in its namesake calponin in smooth muscle is not yet defined. Study of the function of SMTNx and its CHD will have to take into account its variability in sequence, structure, and expression. The SMTN E20+ variant present in non‐muscle cells encodes an extended C‐terminus tail with a potential ubiquitination motif, perhaps so that already transcribed SMTN mRNA and already translated protein can be rapidly degraded as cytoskeleton turns over during SMC de‐differentiation and proliferation. SMTN E20− and SMTNL2 contain a highly homologous helix VI of CHD2 except for an 8 amino acid extension in the former. This suggests that they may serve similar functions in smooth and striated muscle, respectively, where they are highly expressed, with the C‐terminal extension giving an added function to the CHD2 specific to smooth muscle. We did not detect any special attributes of the amino acid composition of these CHD2 variants that would suggest a specific unique function. Interestingly skeletal muscle expresses both CHD2 variants via SMTNL1‐2, suggesting it could have a rapidly turning over pool for cytoskeletal reorganization and a more stable pool conferring SMTNx function.

Global knock‐outs of SMTN and SMTNL1 have produced intriguing phenotypes (Lontay et al., 2010; Niessen et al., 2005; Rensen et al., 2008; Wooldridge et al., 2008) (reviewed in Murali & MacDonald, 2018). Yet tissue‐specific function cannot be inferred from these germline (global) knockout experiments. The precise function of the variable CHD2 containing SMTN family members will best be defined by tissue‐specific approaches, including gain/loss/switch‐in‐function experiments combined with cell, molecular, and physiological assays. Whether SMTNL1/2 highly expressed in skeletal muscle serve the same or similar functions as SMTN E20−/+ expressed in smooth/non‐muscle requires experimental testing. While it has been suggested based on results of germline KO experiments that SMTNLs may serve a function in the smooth muscle cytoskeleton (reviewed in Murali & MacDonald, 2018), this seems unlikely given its low expression in relation to SMTN and unfavorable stoichiometry to its substrate actin and other actin‐binding/cytoskeleton proteins. Alternatively, it has been proposed that SMTNL may translocate to the nucleus and bind with (low abundance) transcription factors to function in transcriptional control of muscle and non‐muscle gene programs (Lontay et al., 2015). Whether SMTNx serve the exact same functions in tissues where they are expressed at high (smooth and skeletal muscle) vs low levels seems unlikely but requires experimental testing. It is also intriguing to note that cardiac muscle is the only muscle type in which none of the SMTN family members are expressed, suggesting that a different cytoskeletal protein may fill that role.

Variability in proteins' C‐termini through AEU is common and often results in addition, subtraction, or alteration of functional modules and rewiring of signaling networks (Buljan et al., 2012; Minde et al., 2017; Sharma & Schiller, 2019; Yang et al., 2016) reviewed in (Fisher, 2017). CHDs most often occur as pairs and more often at the N‐terminus, so a single C‐terminal CHD2 as in SMTNx is atypical. Indeed, we did not find other examples where alternative exon splicing is used to generate variable CHDs. However, there are several examples of AEU that could affect CHD function: (1) many unique exons in Plectin adjacent to the N‐terminal CHD change actin affinity and binding partners (Wiche, 2021) (2) alternative 1st exons in filaminA near the N‐terminus CHD1/2 domains, mutations in which cause complex human disease with smooth muscle‐dependent chronic intestinal pseudo‐obstruction as one feature (Chung et al., 2021; Jenkins et al., 2018; Zada et al., 2023). In conclusion, variable SMTNx CHD2s coupled with tissue‐specific expression suggests isoform‐specific functions that require further study in the cell type‐specific context in which they appear.

## AUTHOR CONTRIBUTIONS

Steven A. Fisher conceived the scientific question, supervised Dhruv Garg in the research, and prepared the manuscript. Dhruv Garg performed the research, prepared the figures, and edited and approved the final version of the manuscript.

## FUNDING INFORMATION

This research was supported by NIH grant R01‐HL066171 and VA MERIT award I01BX004443 to SAF.
